# Comparison of Filtering Methods for Calculating ARFI log(VoA) to Delineate Carotid Plaque Features, In Vivo

**DOI:** 10.1109/ojuffc.2025.3609675

**Published:** 2025-09-12

**Authors:** SHUREED QAZI, KEERTHI S. ANAND, JONATHON W. HOMEISTER, MARK A. FARBER, CATERINA M. GALLIPPI

**Affiliations:** 1 Lampe Joint Department of Biomedical Engineering, The University of North Carolina at Chapel Hill, Chapel Hill, NC 27599 USA; 2 Vadum Inc., Raleigh, NC 27606 USA; 3 Department of Pathology and Laboratory Medicine, The University of North Carolina at Chapel Hill, Chapel Hill, NC 27599 USA; 4 Department of Surgery, The University of North Carolina at Chapel Hill, Chapel Hill, NC 27599 USA

**Keywords:** Acoustic radiation force impulse (ARFI) imaging, atherosclerosis, carotid plaque, FIR filter, generalized contrast-to-noise ratio, IIR filter, principal component analysis (PCA) filtering, stroke, variance of acceleration (VoA)

## Abstract

Carotid atherosclerosis is a major cause of ischemic stroke, and the ability to non-invasively assess plaque composition and structure is critical to effective stroke risk assessment. Carotid plaque components are delineated noninvasively by Acoustic Radiation Force Impulse (ARFI)-derived Variance of Acceleration, evaluated as its decadic log (log(VoA)). To date, this log(VoA) parameter has been calculated by isolating the variance in ARFI-induced displacement profiles using the second-order time derivative (SOTD), a high-pass filtering operation. The purpose of this study was to compare the performance of the SOTD filter to various other filtering methods in application to delineating human carotid plaque components, *in vivo*. Specifically, the SOTD filter was compared to Principal Component Analysis (PCA), Finite Impulse Response (FIR), Infinite Impulse Response (IIR), and mean-center spatial (MCS) filters. Filter performances were evaluated in terms of the resulting log(VoA) generalized contrast-to-noise ratio (gCNR) for distinguishing plaque features in human carotid plaques, *in vivo*, which were validated by spatially aligned histology. Results indicated that the SOTD filter consistently provided the highest gCNR for most plaque components, whereas the performances yielded by the other filters were more variable. The study demonstrated that the SOTD filter remains the preferred method for log(VoA) calculation due to its effectiveness for delineating carotid plaque features.

## I. INTRODUCTION

ACCORDING to the World Health Organization, 15 million people suffer from stroke worldwide each year. These stroke occurrences are fatal to 5 million people, and another 5 million are left permanently disabled [1]. The two major types of strokes are ischemic and hemorrhagic [2]. Most ischemic strokes are caused by atherosclerotic plaque that rupture suddenly and without warning [3]. Therefore, assessing plaque rupture potential is critical to preventing stroke.

Several imaging techniques are routinely employed to assess the rupture potential of carotid atherosclerotic plaque, including duplex ultrasound [4] and X-ray angiography [5]. These methods primarily provide information on plaque size and the degree of blood flow obstruction, or stenosis, but offer limited or no insights into plaque composition or structure. Crucially, rather than degree of stenosis, the composition and structure of plaques are the most important determinants of plaque rupture potential [6], [7].

### A. CAROTID PLAQUE COMPOSITION AND STRUCTURE CONVEY RUPTURE POTENTIAL

A carotid plaque can be broadly categorized into four primary components: calcium (CAL), collagen (COL), lipid-rich necrotic core (LRNC), and intraplaque hemorrhage (IPH) [8], [12]. Among these, high percentage composition of LRNC and IPH is correlated to high rupture potential [6], [26], which increases the likelihood of an ischemic stroke. The thinning or rupture of the collagenous layer, referred to as the fibrous cap, that separates plaque components from luminal blood flow has also been identified as a critical predictor of cerebrovascular events. Specifically, fibrous cap disruption has been associated with a hazard ratio (HR) of 5.93 (95% confidence interval [CI]: 2.65–13.20) for stroke or transient ischemic attack (TIA), while the presence of IPH (HR = 4.59, CI: 2.91–7.24) and LRNC (HR = 3.00, CI: 1.51–5.95) also represent significant risk factors [24], [25].

In addition to composition, plaque rupture potential is influenced by structural characteristics, including the shape, size, and location of calcific deposits. Microcalcifications and spotty calcifications have been linked to inflammatory activity and increased rupture risk, whereas larger, consolidated calcifications are generally considered stabilizing [27]. Furthermore, the presence of multiple or superficial calcific nodules has been associated with IPH and plaque rupture [27].

Therefore, accurate delineation of both the structure and composition of carotid plaques—including the presence, size, and distribution of COL, IPH, LRNC, and CAL—is essential for assessing stroke risk [6], [7], [15].

Current methods for clinically assessing the structure and composition of carotid plaques face multiple limitations. For example, X-ray computed tomography can distinguish between calcified and non-calcified plaques but has poor contrast for soft tissues components such as COL, LRNC, and IPH [9], [10], [11]. Angiography can identify stenosis affecting blood flow but cannot differentiate between plaque tissue types. Positron emission tomography, which uses radiolabeled tracers to target regions of high macrophage activity linked to plaque necrosis, suffers from poor structural delineation of plaques and involves exposure to nuclear radiation [10], [11]. Magnetic resonance imaging can visualize plaque morphology, including LRNC, IPH, and the arterial wall, but is costly and has limited availability. Intravascular optical coherence tomography provides high-resolution images of CAL, fibrous deposits, and lipid regions in plaques, but it is invasive and has limited penetration depth [10].

Compared to these methods, transcutaneous ultrasound is advantageous for assessing carotid plaque composition and structure due to its affordability, accessibility, nonionizing nature, and ability to image several centimeters into tissue [9].

### B. VARIANCE OF ACCELERATION IMAGING

Previous studies have demonstrated that the structure and composition of carotid plaques can be noninvasively delineated using Acoustic Radiation Force Impulse (ARFI) imaging. In initial investigations, plaque features were differentiated based on ARFI-induced peak displacement (PD). While PD was shown in vivo to distinguish stiff components, such as CAL and COL, from soft components, such as LRNC and IPH, in patients undergoing clinically indicated carotid endarterectomy, it lacked the specificity to differentiate between soft (IPH vs. LRNC) or between stiff (CAL vs. COL) tissue types.

Given the clinical importance of distinguishing COL from CAL, which is essential for assessing fibrous cap integrity and localizing calcific deposits, and of differentiating IPH from LRNC, due to the higher hazard ratio for stroke associated with IPH, ARFI Variance of Acceleration (VoA) imaging was developed to enhance plaque feature delineation [12], [13], [14], [15].

ARFI Variance of Acceleration, evaluated as its decadic log and denoted, ‘Log(VoA)’, exploits ARF-induced displacement measurement variance to differentiate plaque features [12], [13]. This approach is relevant because, as described by the Cramer-Rao Lower Bound (CRLB) [16], variance in displacement estimates, or ‘jitter’, is inversely proportional to signal correlation, which decreases with increasing displacement, and SNR, which is lower is less echogenic regions. Thus, plaque components that displace farther in response to an ARF excitation and are less echogenic (like IPH and LRNC) yield higher displacement-tracking variance than components that displace less and are more echogenic (like COL and CAL). For a more comprehensive explanation, the reader is referred to Torres et al. [12], [13], and Anand et al. [15].

In our prior work, ARFI-induced displacement estimation variance was accentuated and isolated by taking the second-order time derivative (SOTD), a high-pass filtering operation, and calculating the average variance over a measurement kernel. However, alternative filtering approaches may achieve better jitter isolation and, thus, improve delineation of plaque components [13]. The purpose of this study was to compare displacement profile filters in terms of the separability of human carotid plaque features in the resulting *in vivo*log(VoA) images, as validated by spatially aligned histology.

## II. METHODS

### A. ARFI IMAGING

In an IRB-approved clinical study, ARFI imaging was performed using a Siemens S3000 scanner equipped for research purposes and a 9L4 transducer (Siemens Healthineers, Ultrasound Division, Issaquah, WA). The ARFI excitations were 300 cycles in duration and centered at 4.21 MHz. Tracking pulses were 2 cycles in duration and centered at 6.15 MHz, a pulse repetition frequency of 10 kHz. Excitations were delivered, and induced displacements tracked, in 40 evenly spaced lateral positions across a 10-mm lateral range. In each lateral position, two reference tracking lines were acquired, an ARFI excitation was transmitted, and then an ensemble of 40 tracking lines was collected.

A skilled sonographer acquired ARFI and B-mode data in the carotid plaques of 17 patients with clinical indication for carotid endarterectomy (CEA). The collection occurred in the pre-surgical holding area immediately before surgery, with the carotid artery positioned in longitudinal view and the surgical plaque approximately centered in B-mode and ARFI image frames. The carotid plaque was identified, and then the sonographer manually swept the transducer elevationally and laterally to acquire ARFI and B-mode data over the plaque volume. These data were later used to aid with image-to-sample registration after plaque extraction. Plaque boundaries were delineated from the ultrasound B-mode images by a sonographer.

### B. DATA PROCESSING AND ANALYSIS

**FIGURE 2.**
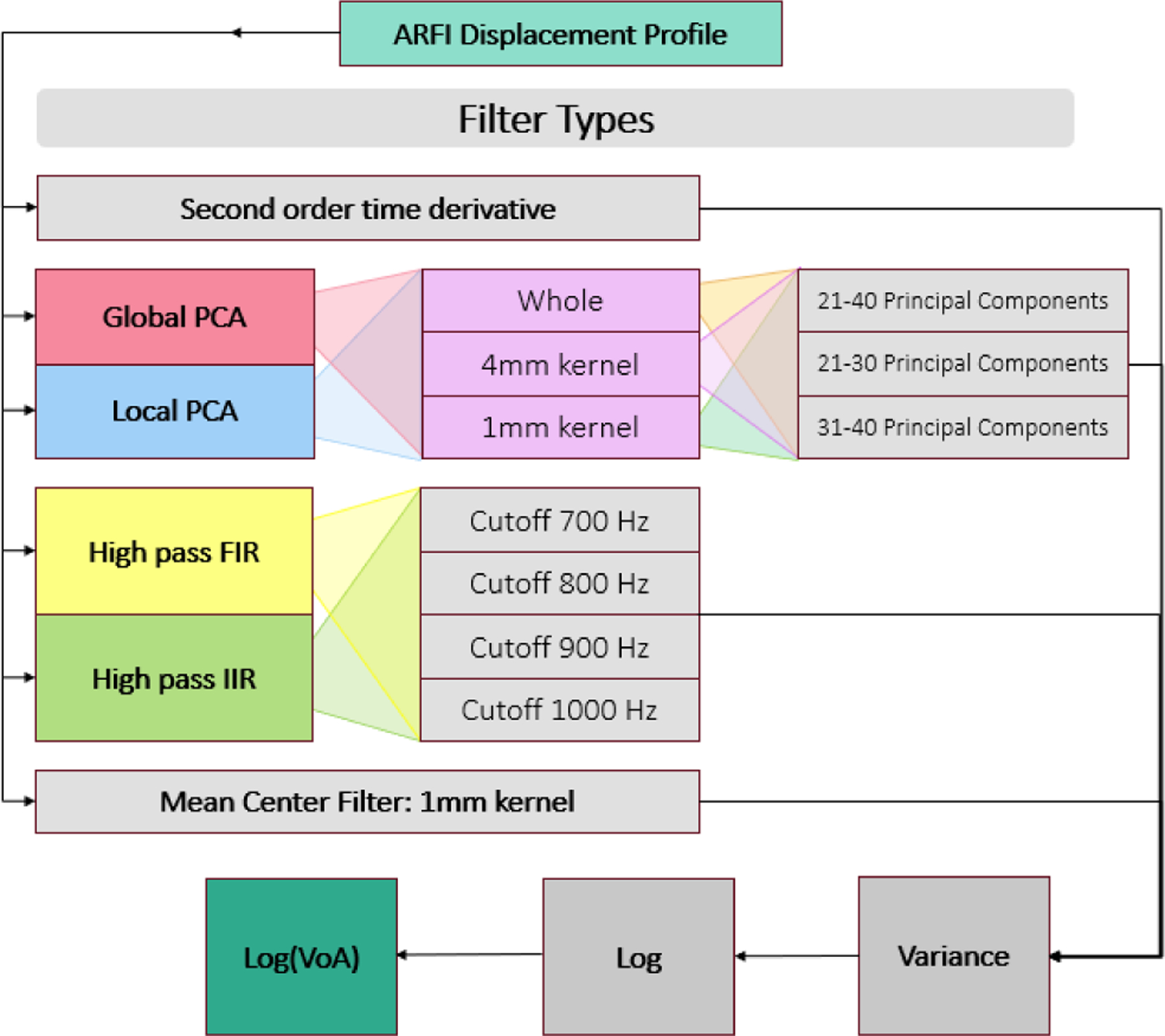
Block diagram illustrating the employed systematic approach to displacement profile filter implementation for ARFI log(VoA) imaging of human carotid atherosclerotic plaque.

First, axial displacements were measured using one-dimensional axial normalized cross-correlation to generate displacement profiles over ensemble time, as described in Pinton et al. [17]. The resulting displacement profiles were then filtered using different approaches, as summarized in Fig. 2 and described in detail below.

### C. SECOND-ORDER TIME DERIVATIVE FILTER

Historically, ARFI log(VoA) has been evaluated by isolating jitter in ARFI-induced displacement profiles using a second-order time derivative (SOTD) filter:

(1) $$y\left(x,y,t\right)=\frac{\delta^{2}d\left(x,y,t\right)}{\delta^{2}t}$$

where $\text{y}\left(\text{x},\text{y},\text{z}\right)$ is the filter output, $\text{d}\left(\text{x},\text{y},\text{z}\right)$ is the ARFI displacement profile through ensemble time, t, located a position x axially and y laterally. Note that, in this implementation, the SOTD is used as a high-pass filtering operation to accentuate and isolate the variance in displacement measurements. The SOTD is not implemented to derive acceleration, *per se*.

### D. PRINCIPAL COMPONENT ANALYSIS FILTERS

Regression filters in the form of principal component analysis (PCA) filters [18], [19], [20], were also applied to the clinical ARFI data. The PCA basis vectors were derived from the ARFI displacement profiles using two approaches: 1) ‘Local PCA’, whereby the basis vectors, or Principal Components (PCs), were derived from displacement profiles measured in the specific plaque being evaluated, and 2) ‘Global PCA’, whereby the basis vectors were generated using displacement profiles from all 17 imaged plaques. For both Local PCA and Global PCA, the basis vectors were calculated from a single kernel that encompassed the entire plaque, a moving 4-mm x 4-mm kernel, or a moving 1-mm x 1-mm kernel.

Once the PCs were generated, those spanning the viscoelastic recovery subspace (as indicated by their smooth temporal projections) were excluded. In contrast, PCs spanning the jitter subspace (characterized by highly variable temporal projections) were retained. Specifically, PCs 21–40, which accounted for the bottom 1% of energy, were identified as spanning jitter. To evaluate the impact of different subspace selections, displacement profiles were projected onto PCs 21–40, 21–30, and 31–40 [18], [20].

### E. FIR AND IIR FILTERS

High-pass Finite Impulse Response (FIR) and Type I Chebyshev Infinite Impulse Response (IIR) filters were implemented in MATLAB (Mathworks Inc., Natick, MA) using simple functions from the *Signal Processing Toolbox*. The specific MATLAB functions used were *designfilt* for creating the filters and *filtfilt* for applying them. The FIR filter had an order of 10, and the passband ripple for the IIR filter was set to 1 dB. The cutoff frequencies for these filters ranged from 700–1000 Hz at 100 Hz intervals, with the passband frequency set to 1.25 times the stopband frequency. The range of cutoff frequencies was determined empirically by analyzing the Fourier transforms of randomly selected displacement profiles and observing the range of frequencies at which the spectrum power dropped to a constant level. These filters were applied to the ARFI displacement profiles at each pixel.

### F. MEAN-CENTER SPATIAL FILTER

A mean-center spatial (MCS) filter was implemented, where the mean displacement profile within a 1mm x 1mm kernel centered about a given pixel was calculated. This mean profile was then subtracted from the original displacement profile at that pixel. Assuming that the tissue response to ARF excitation within the small spatial averaging kernel was consistent, the resultant difference profile would remove the tissue response while generally preserving variance.

### G. VoA CALCULATION

After each filter was applied to the clinical ARFI data per pixel, the variance of the resultant filtered profiles was taken, as described by equation (2) [13], [14], [15]:

(2) $$\begin{array}{l} \log\left(VoA\left(x,y\right)\right)\\ =log_{10}\frac{1}{N-1}\sum_{t=1}^{N}\left|{\left(A\left(x,y,t+\Delta \right)-\mu \left(x,y\right)\right)}^{2}\right| \end{array}$$

Here, N is the calculation kernel length delayed in time by Δ after the occurrence of peak displacement, A is the filtered displacement profile, and μ is the mean of the filtered profile across the kernel. This allows for independent calculation of log(VoA) for every pixel in the image. After previous empirical testing by Torres et al. [13], N and Δ were chosen to be 5 and 20 temporal samples, respectively, corresponding to 0.5 and 2.0 ms. All calculations of log(VoA) were made using custom software written in MATLAB [12], [15].

### H. HISTOLOGICAL VALIDATION

Spatially aligned histology of excised plaque specimens was used as the ground-truth reference for validating the log(VoA) images generated by each filter. Plaques were surgically excised *en bloc* in accordance with standard carotid endarterectomy (CEA) protocols at the University of North Carolina at Chapel Hill. Following excision, samples were fixed in 10% formalin for 24 hours and subsequently imaged using 3D micro-computed tomography (micro-CT) at a voxel resolution of 20*µ*m.

To identify the two-dimensional (2D) imaging plane corresponding to the ARFI acquisition, the micro-CT volumes were compared to pre-operative B-mode images acquired through elevational and lateral sweeps. Anatomical landmarks, including the distance from the carotid bifurcation, the morphology of the plaque-lumen boundary, and the location of echogenic calcium deposits, were used to guide this alignment.

Tissue samples were embedded in an orientation that enabled sectioning along the identified 2D plane. The resulting histological sections were compared to the corresponding micro-CT slice to confirm alignment. If misalignment was observed, the sample was re-embedded and re-sectioned.

After proper sectioning, tissues were stained with hematoxylin and eosin (H&E), Von Kossa (VK) for calcium, and combined Masson’s elastin (CME) for collagen. Stained sections were imaged via microscopy and annotated by a pathologist experienced in atherosclerosis to identify regions of IPH, LRNC, COL, and CAL.

The annotated histology images were then spatially aligned with the B-mode and log(VoA) images using anatomical landmarks. This methodology for histology-based validation of PD- and log(VoA)-derived plaque components is consistent with approaches described in [12], [13].

### I. PERFORMANCE EVALUATION

Once the histology and log(VoA) images were aligned, approximately 0.5 mm × 0.5 mm regions of interest (ROIs) were selected at the center of each identified plaque component to support performance evaluation. If multiple instances of the same component type (e.g., multiple calcium deposits) were present within a single plaque, the ROI was placed in the largest instance of that component.

To minimize edge effects and potential misalignment errors, ROIs were centrally positioned and limited in size. For features smaller than 0.5 mm × 0.5 mm, the ROI size was reduced to match the feature dimensions in the log(VoA) image.

Because the number and size of ROIs varied across plaque components, the total number of log(VoA) values per component also varied. To ensure class balance, a random subsampling approach was applied to equalize the number of log(VoA) values across components, resulting in 668 unique values per class.

The distribution of these values was compared across plaque components using the generalized contrast-to-noise ratio (gCNR) [21], [22] and the Kruskal–Wallis test. The gCNR metric was selected for its ability to quantify contrast between qualitative distributions, such as log(VoA). A gCNR value of 0 indicates complete histogram overlap (i.e., indistinguishable distributions), while a value of 1 indicates no overlap (i.e., fully separable distributions) [22]. The gCNR calculation is given in equations (3) and (4), where $p_{i}$ and $p_{o}$ denote the two distributions of interest, and *ε*_o_ denotes the region of intersection [22]:

(3) $$OVL=\int_{-\infty }^{\varepsilon_{0}}p_{i}\left(x\right)dx+\int_{\varepsilon_{0}}^{\infty }p_{o}\left(x\right)dx$$

(4) gCNR=1−OVL

In addition to the gCNR metric, the Kruskal–Wallis test was used to evaluate statistically significant differences in the distribution of log(VoA) values among plaque components, with a significance threshold set at α=0.05.

### J. IMAGE DISPLAY

Log(VoA) images were created using the Jet colormap. The color bar range was defined such that the minimum value corresponded to the first quartile of the CAL distribution, and the maximum value corresponded to the third quartile of the IPH distribution, as determined by the respective filter.

Brackets were added to each color bar to indicate the log(VoA) value ranges most likely associated with CAL, COL, LRNC, and IPH, based on their respective distributions. These thresholds were determined by the midpoints of the third and first quartiles of corresponding components (e.g. the first quartile of CAL and the third quartile of COL gives the threshold for CAL-COL boundary).

Due to the continuous nature of the colormap, log(VoA) values near the boundaries of component ranges were mapped to intermediate colors. For example, values near the lower end of the IPH range (typically rendered in red) or the upper end of the LRNC range (typically rendered in yellow) appeared as orange.

## III. RESULTS

**FIGURE 3.**
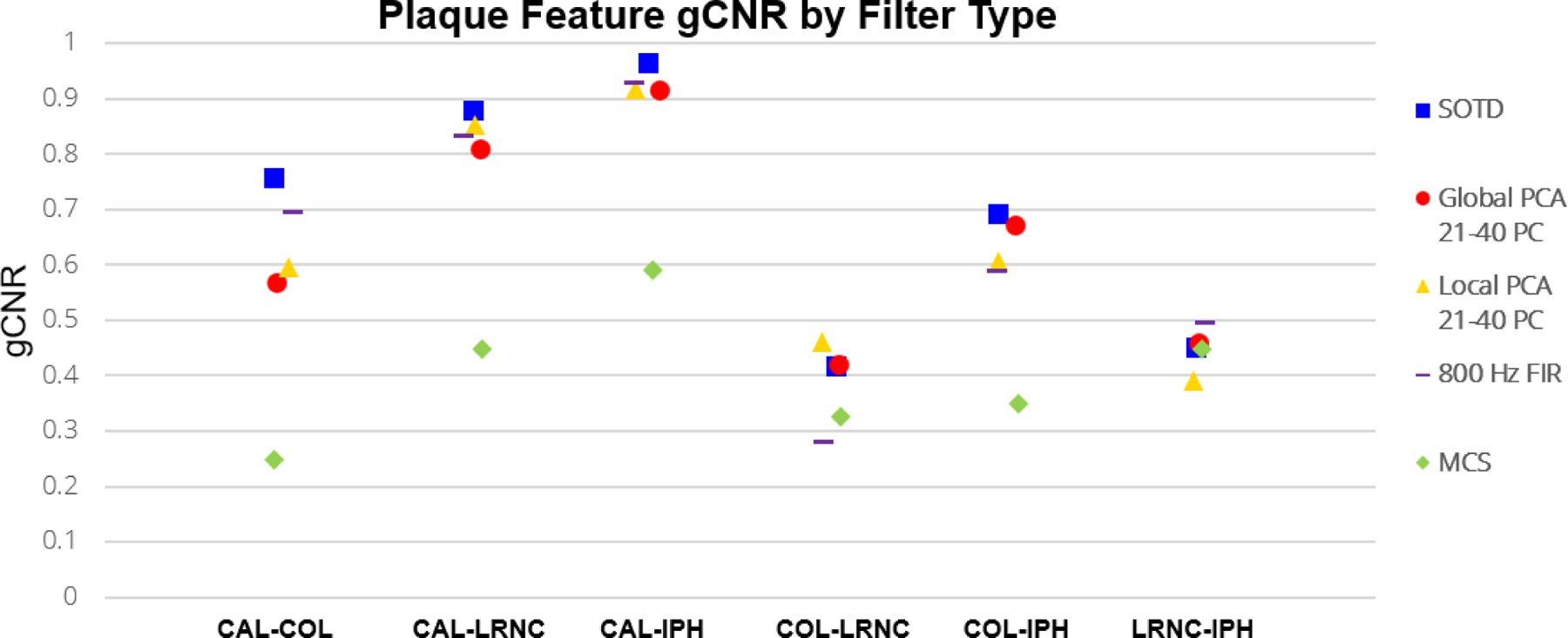
Evaluated over all 17 human carotid atherosclerotic plaques imaged in vivo, gCNR for distinguishing regions of CAL from COL (CAL-COL), CAL-LRNC, CAL-IPH, COL-LRNC, COL-IPH, and LRNC-IPH. Results are shown for only the filter type achieving the highest gCNR within each category of filter. Shape indicates filter type.

Figure 3 shows the gCNR values of log(VoA) measurements obtained using different filters for discriminating between plaque components, as evaluated across all 17 *in vivo* human carotid plaques. For visual clarity, only results for the filter types achieving the highest gCNRs within each category of filter are shown; comprehensive gCNR results for all filters are provided in Supplemental Table 1. Among the Global PCA implementations, the best performance was achieved by the version that derived PCs from a single kernel encompassing the entire plaque and retained PCs 21–40 to isolate jitter. Similarly, the most effective Local PCA filter implementation also used a single plaque-wide kernel and retained PCs 21–40. For frequency-based high-pass filters, the FIR filter with an 800 Hz cutoff frequency yielded the highest gCNR.

**FIGURE 4.**
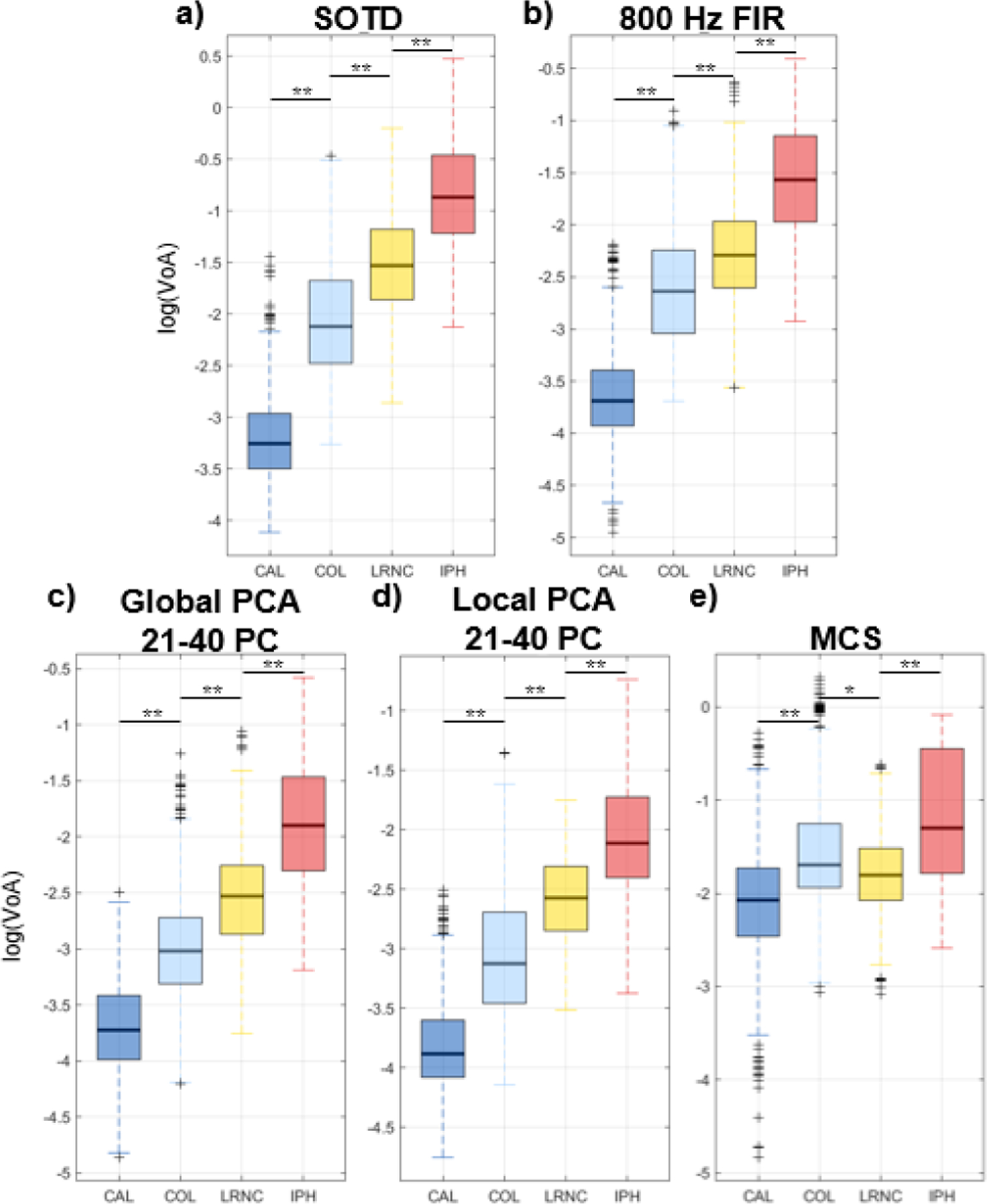
Evaluated over 17 human carotid atherosclerotic plaques imaged *in vivo*, box plots showing the distribution of log(VoA) values within histologically-validated regions of CAL, COL, LRNC, and IPH. Results are shown for the filter type achieving the highest gCNR within each category of filter. Filter types: (a) SOTD (b) 800 Hz high-pass FIR (c) Global PCA using a single kernel and retaining PCs 21–40, (d) Local PCA using a single kernel and retaining PCs 21–40, and (e) MCS. Statistical difference (Kruskal-Wallis, p*<*0.05) indicated by ‘∗” and statistical difference (p*<*0.001) indicated by ‘∗∗’.

Figure 4 presents box plots illustrating the distributions of log(VoA) values in regions identified as CAL, COL, LRNC, and IPH. Each box represents the first, second (median), and third quartiles of the distribution, while the whiskers indicate the maximum and minimum values, excluding outliers. Consistent with Figure 3, results are shown for the filters that achieved the highest gCNR in each category. Statistically significant differences in log(VoA) distributions were observed between plaque components for all filters except MCS. Notably, for all filters except MCS, log(VoA) values exhibited a consistent increasing trend from CAL to COL to LRNC to IPH. Based on the data shown in Figure 4, log(VoA) thresholds for plaque component identification were established as reported in Table 1.

**FIGURE 5.**
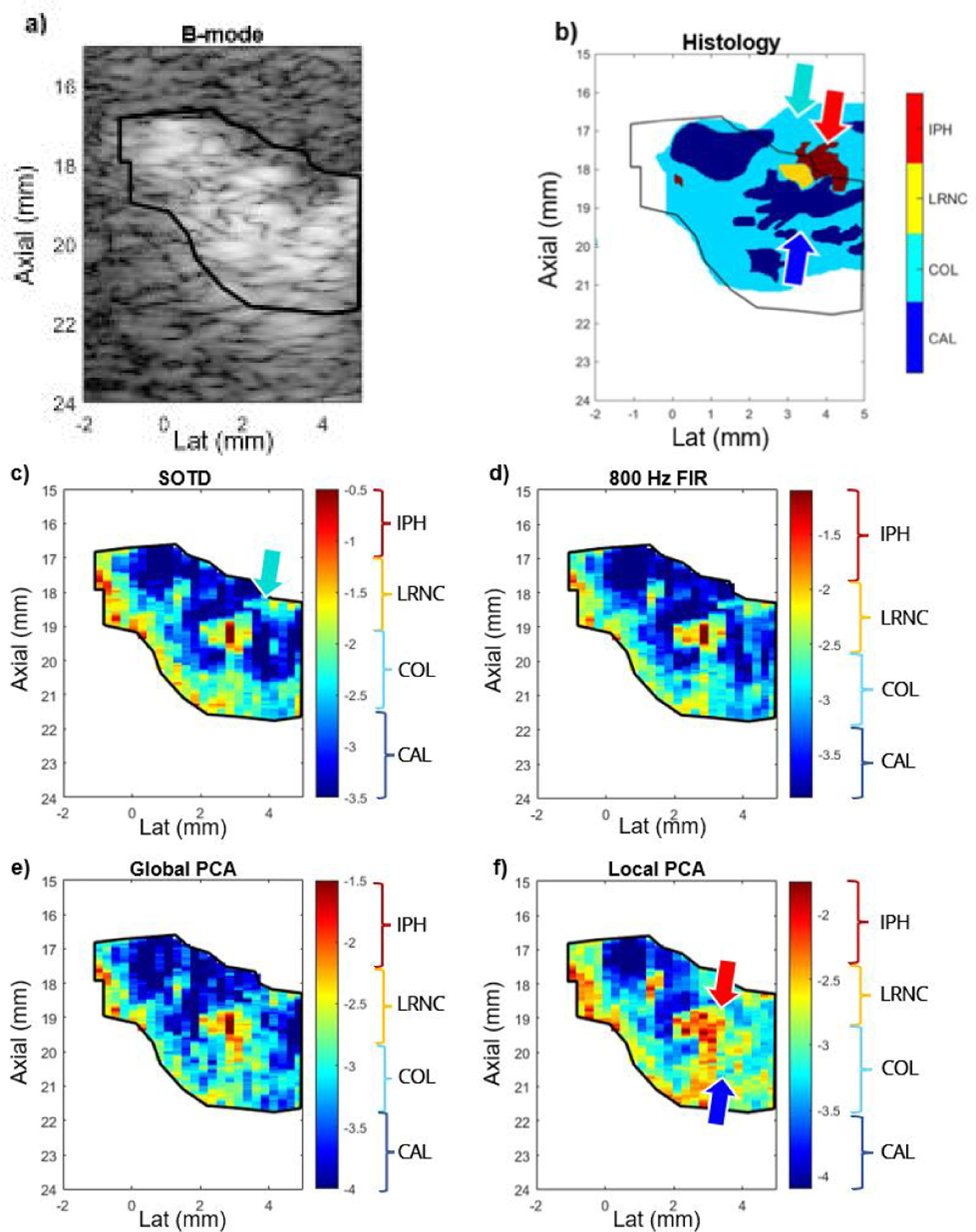
In a representative carotid atherosclerotic plaque in a 62-year-old male CEA patient, (a) *in vivo*B-mode and (b) spatially aligned, color-blocked histology image, where color blocking indicates the pathologist-delineated plaque features, red arrow points to a region of IPH and LRNC, and blue arrow points to a surrounding region of CAL. Spatially corresponding log(VoA) images generated by using the (c) SOTD, (d) 800 Hz FIR, (e) Global PCA, and (f) Local PCA displacement profile filters. Red and dark blue arrows point to regions where the Local PCA filter yielded a different representation of plaque composition than the other filters. The cyan arrow points to a region of clinical interest in the SOTD log(VoA) image, a COL region which constitutes a fibrous cap.

Figure 5 shows, for a representative carotid artery plaque imaged *in vivo* in a 62 year-old male CEA patient, spatially aligned B-mode, histology, and parametric log(VoA) images created using the SOTD and the FIR, Global PCA, and Local PCA filters that achieved the highest gCNR. Recall that the brackets added to the color bars indicate the log(VoA) value ranges most likely associated with CAL, COL, LRNC, and IPH, based the log(VoA) thresholds in Table 1. The B-mode shows hyperechogenic regions that coincide with focal calcium deposits in the histology. The histology also shows that the calcium deposits are positioned below and beside a focal region of LRNC and IPH. The log(VoA) images are generally consistent in their depictions of CAL, LRNC, and IPH features, but with some variations, highlighted by arrows.

**FIGURE 6.**
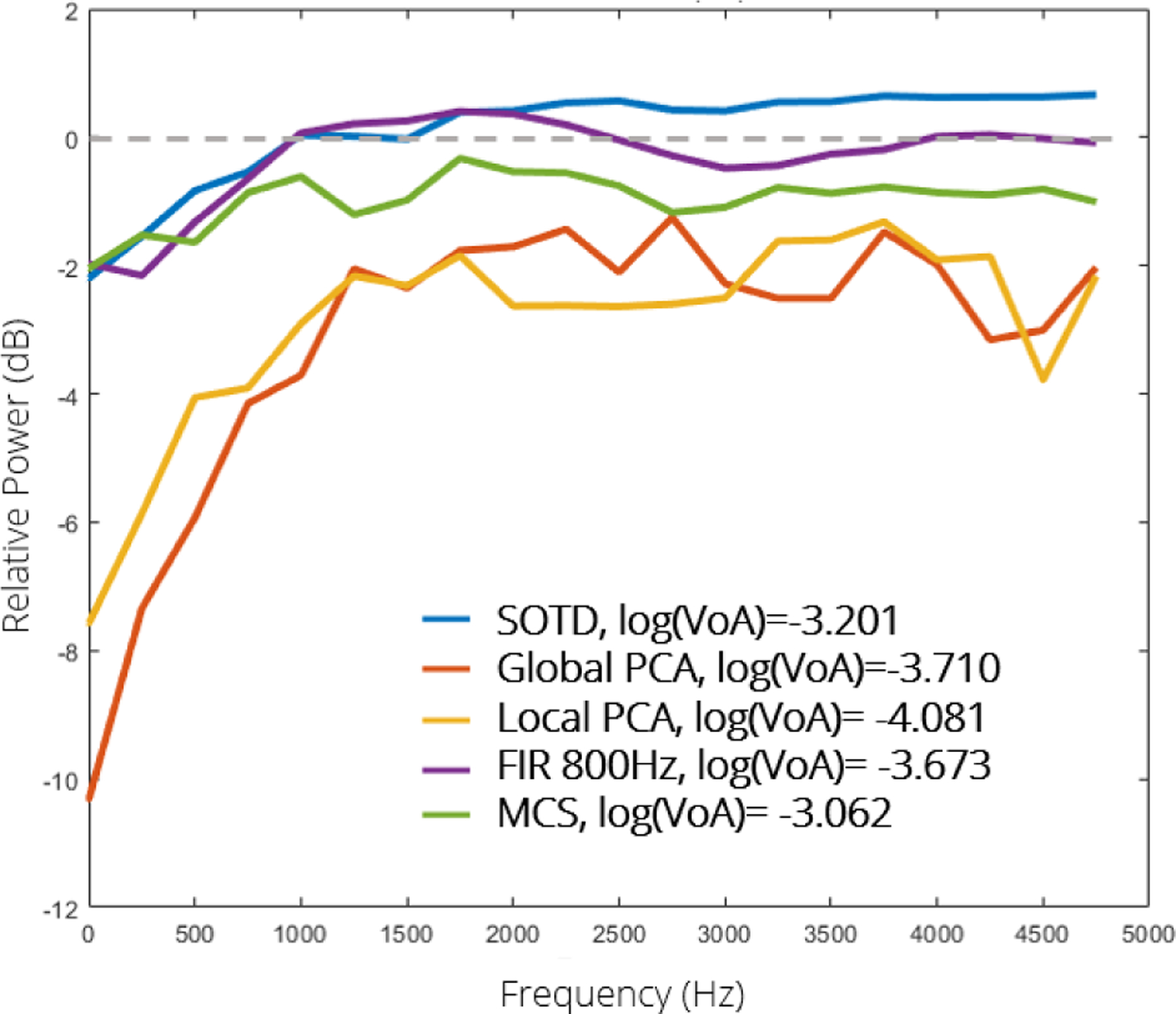
Relative power spectra of a representative displacement profile from a region of CAL after filtering as indicated in the legend. The spectra are normalized by the corresponding unfiltered spectrum.

To further evaluate filter performance differences, Fig. 6 shows the power spectra of a representative filtered displacement profile from a region of CAL, normalized by the power spectrum of the unfiltered displacement profile.

## IV. DISCUSSION

The results presented herein demonstrate that the effectiveness of ARFI Log(VoA) imaging for delineating the structure and composition of *in vivo* human carotid atherosclerotic plaque is influenced by the choice of jitter isolation filter. As shown in Fig. 3, the SOTD filter consistently yielded among the highest gCNR values for discriminating the four carotid plaque components, with its largest advantage observed for delineating regions of CAL from COL. Notably, the SOTD filter performed more comparably to other filters when delineating LRNC from COL and from IPH. These results suggest that the SOTD filter offers enhanced discrimination of low-amplitude jitter, such as that expected in stiff and echogenic CAL.

Figure 3 (and Supplemental Table 1) also reveal a notable trend across all filters: the gCNR for CAL discrimination increases progressively as CAL is compared against COL, LRNC, and then IPH, with CAL-IPH discrimination approaching gCNR values near 1.0. Similarly, the gCNR for COL discrimination increases as COL is compared to LRNC and then IPH. These trends align with the expected increasing differences in jitter magnitude across these plaque components. Despite subtle performance variations, the results indicate that all implemented filters reasonably isolate jitter from ARFI-induced displacement profiles for log(VoA) imaging. One exception is the MCS filter, which performed notably worse than the other filters in discriminating all plaque components except LRNC-IPH. This suggests that the MCS filter’s utility may be limited to extracting high-amplitude jitter, making it less relevant for applications requiring finer jitter discrimination.

The gCNR trends described above can be further interpreted by examining the distribution of log(VoA) values within each identified plaque component, as shown in Fig. 4. Among the five filter types evaluated, the SOTD, 800-Hz FIR, Global PCA, and Local PCA filters produced log(VoA) distributions that were statistically distinct across all four plaque components. Notably, across all filters, the largest effect sizes were observed between CAL and COL, suggesting that CAL is more reliably delineated than other components. In contrast, the smallest effect sizes were found between COL and LRNC, indicating that LRNC may be the most challenging to distinguish. To further validate these findings, future work will include a statistical reader study and receiver operating characteristic (ROC) analysis for log(VoA)-based plaque component detection, similar to prior evaluations conducted for ARFI peak displacement imaging [23].

Figure 5 provides further validation of the SOTD filter’s superior performance by demonstrating its ability to achieve enhanced CAL discrimination in a representative human carotid atherosclerotic plaque, in vivo. While all four filters generated log(VoA) images that delineate CAL, LRNC, and IPH features within the plaque, the delineation achieved using the SOTD filter most closely aligns with histological findings. For example, the CAL deposits to the right of the IPH region are accurately identified in the SOTD log(VoA) image, whereas the 800-Hz FIR and Global PCA filters indicate the CAL in this region with progressively less distinction from the background COL. The Local PCA filter fails to capture the CAL deposits to the right of the IPH region, underscoring the SOTD filter’s superior performance for this specific feature. These results are consistent with the high gCNR for CAL-COL discrimination achieved by the SOTD filter, as shown in Fig. 3. Additionally, Figure 5 shows comparable delineation of the IPH region by the SOTD, 800-Hz FIR, and Global PCA filters. However, the Local PCA filter overestimates the size of the IPH feature. This finding aligns with the lower gCNR for COL-IPH discrimination achieved by the Local PCA filter compared to the SOTD and Global PCA filters. Interestingly, the 800-Hz FIR filter performed better for IPH delineation than its gCNR values might suggest.

From a broader clinical perspective, log(VoA) imaging, such as the examples shown in Fig. 5, holds promise for assessing the structure and composition of *in vivo* carotid plaques to support stroke risk stratification. For instance, in the SOTD-derived log(VoA) image shown in Fig. 5(c), the identification of LRNC and IPH beneath a COL layer, or fibrous cap (denoted by the cyan arrow), suggests a rupture-prone plaque phenotype. Such a configuration may indicate the need for CEA or alternative clinical intervention to mitigate stroke risk.

Figure 6 offers insights into the variable performances observed in terms of gCNR and plaque feature delineation. While all relative power spectra confirm the high-pass nature of the implemented filters, only the SOTD filter maintained relative power above 0 dB for frequencies greater than 1500 Hz. This suggests that the SOTD filter effectively amplifies jitter in the displacement profile, contributing to its enhanced performance for CAL discrimination, where jitter magnitude is expected to be lower. In contrast, the Global and Local PCA filters achieved greater low-frequency suppression but at the expense of reduced high-frequency amplitude. This tradeoff likely explains why the PCA filters were outperformed by the SOTD filter; the benefits of improved high-frequency jitter isolation were offset by an overall reduction in filtered profile magnitude.

While the results indicate that the SOTD filter provided the highest overall separability of human carotid plaque features using *in vivo* log(VoA) imaging, several factors may have influenced the study outcomes. One key limitation was the imperfect alignment between *in vivo* log(VoA) images and histological sections, primarily due to plaque deformation during surgical excision and tissue processing. This misregistration could have affected gCNR measurements and comparisons of log(VoA) distributions across plaque components. However, this risk was mitigated by excluding boundary regions when selecting component ROIs. A second limitation was that CAL and COL components were generally larger in size compared to LRNC and IPH components. As a result, regions of interest (ROIs) placed within LRNC and IPH were more likely to include boundary regions of the features, increasing the likelihood of errors arising from edge effects and misalignment with histological reference data. A third limitation involved shadowing artifacts caused by large CAL deposits, which obscured deeper plaque structures. As a result, the effective sample size for all plaque components, including CAL, was reduced after class balancing. Notably, this suggests that in plaques with substantial CAL burden, log(VoA) imaging may fail to detect components in shadowed regions that could contribute to stroke risk. Addressing these shadowing artifacts through improved data acquisition and image processing techniques may enhance the accuracy of plaque delineation using log(VoA) imaging.

These results suggest that log(VoA) imaging using the SOTD filter enables spatially precise discrimination of focal plaque components. Carotid plaque characterization has also been performed using alternative ultrasound elasticity imaging methods. For example, Marveli et al showed that shear wave elastography (SWE)-derived group and phase velocities correlated with MRI-identified features that suggested carotid plaque vulnerability [28]. Also, Li et al. reported that Pulse Wave Imaging (PWI) and strain imaging measured higher compliance in lipid-rich plaque [29], with Karageogos et al.showing that wall heterogeneity indicated plaque vulnerability [30]. Cloutier et al described that ultrasound elastography combined with echogenicity analysis distinguished symptomatic from asymptomatic plaques, with Roy-Cardinal describing the use of machine learning and quantitative ultrasound features to indicate overall degree of calcium and lipid burden and fibrous cap rupture [31], [32]. Finally, Hansen et al. showed that (fibro)atheromatous plaques exhibit significantly higher radial strain than fibrous plaques [33]. While these studies support that the evaluated methods may be relevant to noninvasive plaque vulnerability assessment, the methods provide general descriptions of overall plaque composition, by measuring mechanical effects of the presence of vulnerable plaque. In contrast, log(VoA) imaging with the SOTD filter provides point-wise plaque feature delineation, mapping the location and size of specific plaque components to better inform stroke risk assessment.

Future research will focus on applying log(VoA) imaging using SOTD filter in larger clinical studies. These studies will aim to correlate the derived plaque composition and structural features with the incidence of stroke, transient ischemic attacks, and other neurological symptoms. Additionally, automated algorithms for plaque component delineation based on log(VoA) values will be developed to streamline and accelerate the clinical interpretation of log(VoA) images. Beyond the carotid applications, future work will also explore the utility of log(VoA) imaging in other vascular beds to assess the method’s broader diagnostic potential.

## V. CONCLUSION

This study demonstrates the superior performance of the SOTD filter for discriminating carotid plaque components using log(VoA) imaging. The SOTD filter achieved the highest gCNR values, particularly for distinguishing CAL from COL, and delineated plaque features most consistently with histological findings. The ability of the SOTD filter to amplify high-frequency jitter while preserving displacement profile magnitude likely underpins its enhanced performance. These results underscore the potential of the SOTD filter as a valuable tool for advancing ARFI log(VoA) imaging of atherosclerotic plaques, offering improved characterization of plaque composition and aiding in clinical decision-making. Future studies should explore its applicability in larger patient cohorts, automate its interpretation, and investigate its performance in other arteries.

## Supplementary Material

supp1-3609675

This article has supplementary downloadable material available at https://doi.org/10.1109/OJUFFC.2025.3609675, provided by the authors.

## Figures and Tables

**FIGURE 1.**
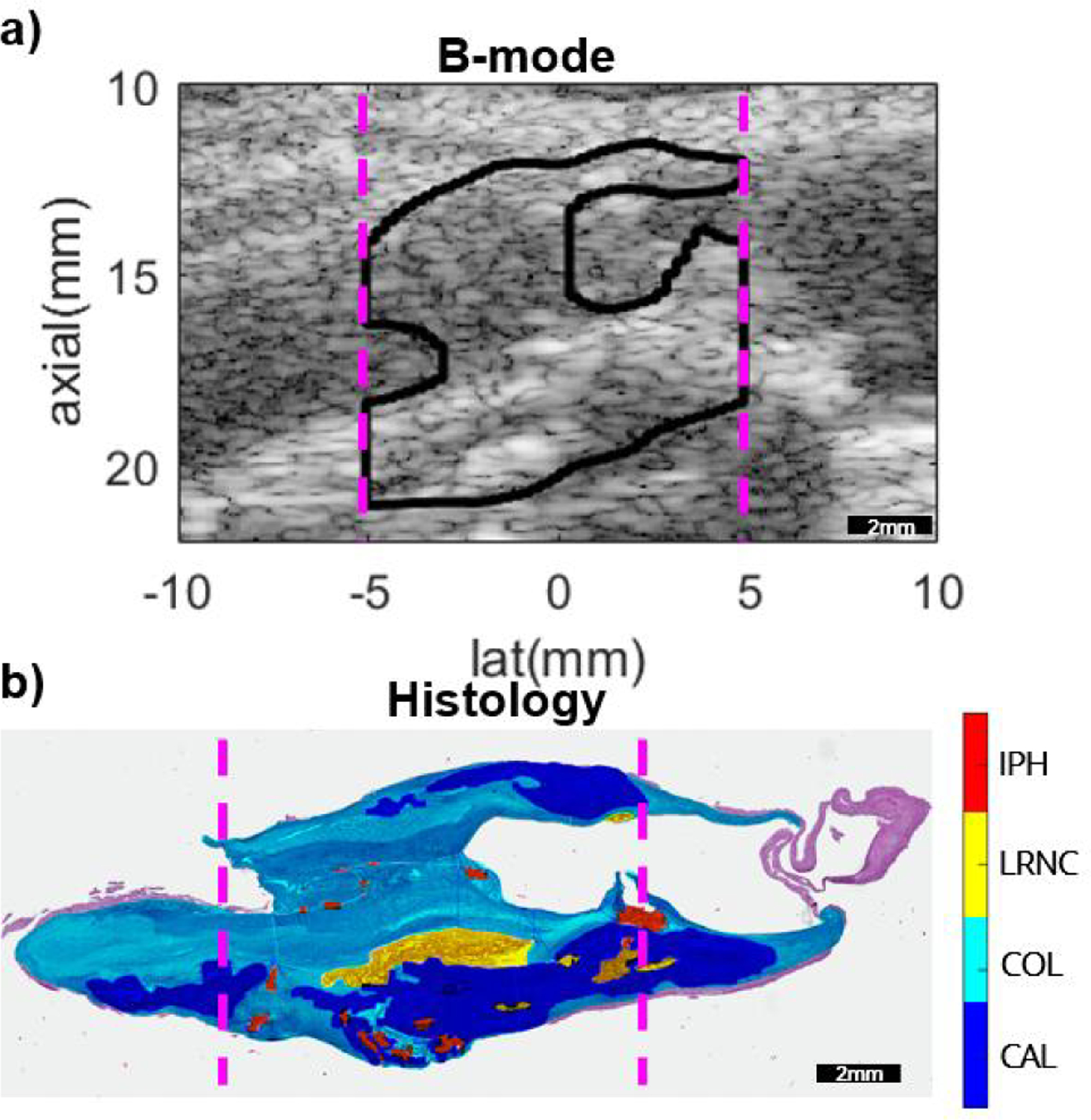
(a) A representative presurgical *in vivo* B-mode image and (b) the corresponding spatially-aligned histology for a human carotid atherosclerotic plaque in a female CEA patient aged 80 years. The dashed vertical lines in both panels indicate the lateral span of ARFI imaging. The color coding in (b) indicates the pathologist-delineated plaque features, as indicated by the color bar. Purple shade is the background stain color, shown in regions left unmarked by the pathologist.

**TABLE 1.** Log(VoA) thresholds for indicating plaque component type for each filtering method.

Log(VoA) Thresholds				
Filter Type	CAL	COL	LRNC	IPH
**SOTD**	<−2.72	(−2.72, −1.77)	(−1.77, −1.20)	>−1.20
**800 Hz FIR**	<−3.22	(−3.22, −2.42)	(−2.42, −1.97)	>−1.97
**Global PCA**	<−3.36	(−3.36, −2.79)	(−2.79, −2.28)	>−2.28
**Local PCA**	<−3.53	(−3.53, −2.77)	(−2.77, −2.36)	>−2.36
