# Supplementary material for: Comparison of Filtering Methods for Calculating ARFI log(VoA) to Delineate Carotid Plaque Features, In Vivo
Source: IEEE Open J Ultrason Ferroelectr Freq Control. Author manuscript; Available in PMC 2025 Nov 20. (PMC12629256; doi:10.1109/ojuffc.2025.3609675)
Supplement: supp1-3609675 [file NIHMS2118515-supplement-supp1-3609675.pdf]

Supplemental Table 1. Evaluated over all 17 human carotid plaques imaged *in vivo,* plaque component gCNRs for all displacement profile filters implemented to calculate ARFI log(VoA). Color denotes feature type as green = CAL, blue = COL, yellow = LRNC, and red = IPH. Color-coding indicates the features being contrasted, such that the columns shaded blue, yellow, and red under the green "CAL" tri-column heading include gCNRs for CAL-COL, CAL-LRNC, and CAL-IPH, respectively.

Filter Type			CAL			COL			LRNC			IPH		
SOTD			0.756	0.879	0.964	0.756	0.417	0.692	0.879	0.417	0.451	0.964	0.692	0.451
Global PCA	Entire Plaque	21-40 EV	0.568	0.809	0.915	0.568	0.42	0.671	0.809	0.42	0.459	0.915	0.671	0.459
		21-30 EV	0.503	0.762	0.901	0.503	0.412	0.667	0.762	0.412	0.437	0.901	0.667	0.437
		31-40 EV	0.529	0.783	0.884	0.529	0.417	0.64	0.783	0.417	0.44	0.884	0.64	0.44
		11-40 EV	0.497	0.719	0.86	0.497	0.342	0.603	0.719	0.342	0.498	0.86	0.603	0.498
		6-40 EV	0.385	0.62	0.728	0.385	0.298	0.487	0.62	0.298	0.499	0.728	0.487	0.499
	1mm Kernel	21-40 EV	0.568	0.809	0.915	0.568	0.42	0.671	0.809	0.42	0.459	0.915	0.671	0.459
		21-30 EV	0.503	0.762	0.901	0.503	0.412	0.667	0.762	0.412	0.437	0.901	0.667	0.437
		31-40 EV	0.529	0.783	0.884	0.529	0.417	0.64	0.783	0.417	0.44	0.884	0.64	0.44
	4mm Kernel	21-40 EV	0.568	0.809	0.915	0.568	0.42	0.671	0.809	0.42	0.459	0.915	0.671	0.459
		21-30 EV	0.503	0.762	0.901	0.503	0.412	0.667	0.762	0.412	0.437	0.901	0.667	0.437
		31-40 EV	0.529	0.783	0.884	0.529	0.417	0.64	0.783	0.417	0.44	0.884	0.64	0.44
Local PCA	Entire Plaque	21-40 EV	0.596	0.854	0.918	0.596	0.462	0.606	0.854	0.462	0.391	0.918	0.606	0.391
		21-30 EV	0.543	0.824	0.911	0.543	0.42	0.634	0.824	0.42	0.426	0.911	0.634	0.426
		31-40 EV	0.568	0.831	0.846	0.568	0.445	0.533	0.831	0.445	0.282	0.846	0.533	0.282
		11-40 EV	0.555	0.805	0.901	0.555	0.382	0.601	0.805	0.382	0.474	0.901	0.601	0.474
		6-40 EV	0.474	0.698	0.802	0.474	0.351	0.503	0.698	0.351	0.516	0.802	0.503	0.516
	1mm Kernel	21-40 EV	0.569	0.75	0.866	0.569	0.289	0.545	0.75	0.289	0.395	0.866	0.545	0.395
		21-30 EV	0.553	0.743	0.86	0.553	0.274	0.532	0.743	0.274	0.396	0.86	0.532	0.396
		31-40 EV	0.556	0.733	0.865	0.556	0.284	0.535	0.733	0.284	0.352	0.865	0.535	0.352
	4mm Kernel	21-40 EV	0.547	0.765	0.906	0.547	0.34	0.588	0.765	0.34	0.333	0.906	0.588	0.333
		21-30 EV	0.544	0.775	0.914	0.544	0.344	0.602	0.775	0.344	0.366	0.914	0.602	0.366
		31-40 EV	0.491	0.696	0.82	0.491	0.278	0.478	0.696	0.278	0.261	0.82	0.478	0.261
High Pass 700 Hz	FIR		0.616	0.794	0.9	0.616	0.27	0.516	0.794	0.27	0.524	0.9	0.516	0.524
	IIR		0.355	0.334	0.387	0.355	0.168	0.204	0.334	0.168	0.231	0.387	0.204	0.231
High Pass 800 Hz	FIR		0.696	0.835	0.932	0.696	0.281	0.59	0.835	0.281	0.498	0.932	0.59	0.498
	IIR		0.282	0.344	0.342	0.282	0.11	0.204	0.344	0.11	0.211	0.342	0.204	0.211
High Pass 900 Hz	FIR		0.653	0.824	0.919	0.653	0.276	0.565	0.824	0.276	0.502	0.919	0.565	0.502
	IIR		0.313	0.348	0.17	0.313	0.11	0.209	0.348	0.11	0.247	0.17	0.209	0.247
High Pass 1000 Hz	FIR		0.667	0.827	0.924	0.667	0.284	0.586	0.827	0.284	0.491	0.924	0.586	0.491
	IIR		0.29	0.286	0.255	0.29	0.104	0.157	0.286	0.104	0.156	0.255	0.157	0.156
MCS			0.249	0.447	0.592	0.249	0.325	0.349	0.447	0.325	0.448	0.592	0.349	0.448
